# Impact of SARS-CoV-2 on Host Factors Involved in Mental Disorders

**DOI:** 10.3389/fmicb.2022.845559

**Published:** 2022-04-04

**Authors:** Raina Rhoades, Sarah Solomon, Christina Johnson, Shaolei Teng

**Affiliations:** Department of Biology, Howard University, Washington, DC, United States

**Keywords:** SARS-CoV-2, mental disorders, depression, schizophrenia, psychosis

## Abstract

COVID-19, caused by SARS-CoV-2, is a systemic illness due to its multiorgan effects in patients. The disease has a detrimental impact on respiratory and cardiovascular systems. One early symptom of infection is anosmia or lack of smell; this implicates the involvement of the olfactory bulb in COVID-19 disease and provides a route into the central nervous system. However, little is known about how SARS-CoV-2 affects neurological or psychological symptoms. SARS-CoV-2 exploits host receptors that converge on pathways that impact psychological symptoms. This systemic review discusses the ways involved by coronavirus infection and their impact on mental health disorders. We begin by briefly introducing the history of coronaviruses, followed by an overview of the essential proteins to viral entry. Then, we discuss the downstream effects of viral entry on host proteins. Finally, we review the literature on host factors that are known to play critical roles in neuropsychiatric symptoms and mental diseases and discuss how COVID-19 could impact mental health globally. Our review details the host factors and pathways involved in the cellular mechanisms, such as systemic inflammation, that play a significant role in the development of neuropsychological symptoms stemming from COVID-19 infection.

## Introduction

Post-acute COVID-19 Syndrome, also known as long-COVID, is a significant concern for global public health. The symptoms of long COVID range from length recovery from organ damage, persistent symptoms lasting up to 6 weeks, to a patient presenting as asymptomatic or experiencing a period of healing only to see a return of symptoms that persist from 3 to 6 months, and even sudden death up to 12 months post-infection (Raveendran and Misra, 2021). Neurological and neuropsychiatric symptoms have also been observed in one-third of patients after COVID-19 infection (Schou et al., 2021). These symptoms include depression, anxiety, cognitive deficits, “brain fog,” and fatigue, which have been reported in conjunction with infection by MERS-CoV and SARS-CoV and previous pandemics such as the Spanish Flu (Schou et al., 2021; Stefano, 2021).

Studies have shown that some coronaviruses can invade the brainstem via a synapse-connected route from the lungs and airways (Zhang et al., 2020). A few central mechanisms have been proposed to explain neurological symptoms related to SARS-CoV-2 infection. The first theory, the “indirect attack theory,” proposes that neurological effects are due to the immune impacts triggered by infections, i.e., the cytokine storm (Wu et al., 2020b). The second suggests that the virus gains entry to the central nervous system (CNS) via the olfactory pathway, or peripheral route, and demonstrated in animal models of encephalitis induced by corona viral infection. The reports of deficits in taste, smell, and psychiatric symptoms following coronavirus infection are consistent with the peripheral route or the olfactory pathway as a route of entry for the SARS-CoV-2 virus (Acharya et al., 2020; Butowt and von Bartheld, 2020). Anosmia and other deficits in sensation are features of several mental disorders, including post-traumatic stress disorder (PTSD), major depression disorder (MDD), SCZ, bipolar disorder (BPD), and neurodegenerative disorders. The third route of entry for SARS-CoV-2 into the CNS involves exosomes. Several studies have noted that the cytokine storm cannot explain CNS damage since the inflammatory markers seen in SARS-CoV-2 cases are less abundant than seen in other cases of a viral infection such as H1N1 influenza. Additionally, the lack of detected viral gene expression in the CNS casts doubt on the hypothesis that the cytokine storm is causing or leading contributor to the neurological damage and neuropsychiatric symptoms seen in some SARS-CoV-2 cases. Exosomes have been previously reported to aid in viral pathogenesis (Estrada, 2021).

Viral entry of the coronavirus is mediated by the spike (S) protein, which has two subunits, S1 and S2. The S1 component binds to the host cell receptor, and the S2 subunit mediates the fusion of the virus with the host’s cell membrane. The key to the entry of the SARS-CoV-2 virus into host cells is the angiotensin-converting enzyme 2 (ACE2) receptor, which is expressed in many tissues, including the respiratory system and neurons, and brain endothelium (Hamming et al., 2004; Sheraton et al., 2020). However, viral entry is also dependent on the priming of the S protein by host proteases such as transmembrane serine protease 2 (TMPRSS2) and FURIN. Several virion components linked to the pathology of coronaviruses have also been previously linked to mental health disorders. Coronavirus proteins such as the envelope (E) and nucleocapsid (N) proteins have also been demonstrated to bind to post-synaptic density-95 (PSD-95) and retinoic acid-inducible gene-1 (RIG-1) proteins. The envelope protein of SARS CoV-2 has also been reported to have a PSD-95 binding motif. PSD-95 is a scaffolding protein that plays an essential role in excitatory neurons and viral pathogenesis (Javier and Rice, 2011). Previous investigations have shown that the N protein of SARS-CoV-1, 90% similar to that of SARS-CoV-2, halts cell cycle progression *in vitro* (Li et al., 2005a,b; Dutta et al., 2020). Additionally, the SARS-CoV-2 N protein has been shown to possess a RIG-1 binding domain and inhibit RIG-1-like pathways (Oh and Shin, 2021). Rig-1 is a gene that recognizes viral infection, such as in *Toxoplasma gondii*. The Rig-1 gene has also been found to be associated with schizophrenia (SCZ) diagnosis (Carter, 2009). Additionally, the N-protein activates the cyclooxygenase-2 (COX-2) promoter. Thus, it plays a role in increased inflammation associated with coronavirus infection (Yan et al., 2006). Host receptor ACE2 serves as the point of entry for SARS-CoV-2 via the attachment of the S glycoprotein (Krassowski et al., 2018). A genome-wide association study of 1980 patients infected with SARS-CoV-2 found two loci 3p21.31 and 9q34.2 with genome-wide significance to be associated with severe symptoms. The significant association at the 3p21.31 locus was driven by solute carrier family 6 member 20 (SLC6A20), leucine zipper transcription factor-like 1 (LZTFL1), C-C chemokine receptor 1 (CCR1), FYVE coiled-coil domain-containing protein 1 (FYCO1), CXC motif chemokine receptor 6 (CXCR6), and X-C motif chemokine receptor 1 (XCR1), and the gene contributing to the significant association in the 9q34.2 locus was the histo-blood group ABO system transferase (ABO) (Severe Covid-19 GWAS Group et al., 2020). Additionally, five genes that seem to facilitate infection of the SARS-CoV2 virus are glycogen synthase kinase 3 beta (GSK-3β), furin protease, TMPRSS2, a disintegrin and metalloprotease 17 (ADAM17), and neuropilin-1 (Heurich et al., 2014; Cantuti-Castelvetri et al., 2020; Coutard et al., 2020; Nowak and Walkowiak, 2020).

With this in mind, we must now consider how these viral pathways can activate mental health disorders, as links between infectious disease and mental health disorders have been previously reported. Increased risk of developing SCZ, for example, has been linked to several contagious agents such as *Chlamydia* spp., *T. gondii*, Human Herpesvirus, and Cytomegalovirus (Arias et al., 2012). Coronavirus infection could lead to injury and inflammation, the exacerbation of neuropsychiatric symptoms. Studies of the olfactory epithelia have demonstrated its utility in studying psychiatric disorders as well as neurodevelopmental processes. Deficits in olfactory functioning have been reported in depression and other affective disorders (Taalman et al., 2017; Kamath et al., 2018). Therefore, several proteins affected by coronavirus infection, such as ACE2 and dipeptidyl peptidase 4 (DPP4), are enriched in the epithelia of the respiratory tract (Hamming et al., 2004; Jia et al., 2005; Solerte et al., 2020). Previous work has also reported several genes that may be related to increased susceptibility or resistance to SARS-CoV-2 infection (Wei et al., 2021). And there are several suggested mechanisms by which SARS-CoV-2 may affect the CNS, such as “viral encephalitis, systemic inflammation, organ dysfunction, and cerebrovascular change” (Heneka et al., 2020). This suggests that investigating genes enriched in the respiratory tract or found to be important in SARS-CoV-2 infection may help to understand how coronavirus infections may impact mental health (Wei et al., 2021).

Depression is among the top five leading causes of disability worldwide. Mental health disorders have a significant impact on the global economy, costing as much as 2.5 trillion dollars per year and rising (GBD 2016 Disease and Injury Incidence and Prevalence Collaborators, 2017; The Lancet Global Health, 2020). Therefore, particularly imperative to understand how infectious diseases might be converging with social, economic, and life stressors that are perturbed during global pandemics. Fear, social isolation, anxiety, sleep disturbances, unemployment, and housing insecurity can compound ongoing or predisposed mental health issues. For example, it has been reported in Wuhan, China, that more than half of the residents experienced symptoms of depression and or anxiety (Clemente-Suárez et al., 2021). Many recovered COVID-19 patients have been reported to experience neurological symptoms such as parkinsonism, intracranial hemorrhaging, and strokes. Long-term psychological symptoms such as dementia, anxiety, and psychosis have also been reported (Taquet et al., 2021a). A retrospective cohort study of 62,354 patients showed that hazard ratios for psychiatric diagnoses were higher than influenza, skin infections, and respiratory tract infections for the first 14–90 days following COVID-19 diagnosis (Taquet et al., 2021b). In a retrospective study of 236,379 patients, the authors found that the incidence of neurological and psychological symptoms in the 6 months following COVID-19 diagnosis was between 33 and 62%. Many of these patients were diagnosed with these symptoms for the first time, with an estimated incidence of 1–84% (Taquet et al., 2021a). Additional studies have found that patients with long-COVID have exhibited imbalance, vertigo, hallucinations, headaches, memory deficits, and depression (Mehandru and Merad, 2022).

**FIGURE 1:**
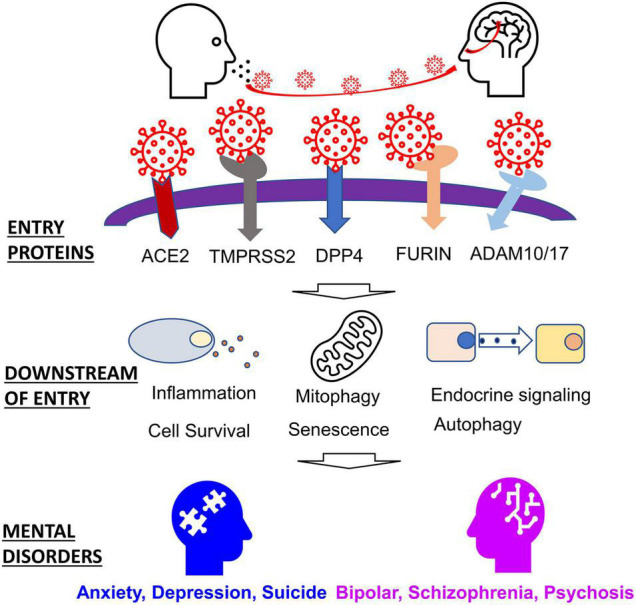
Host factors involved in SARS-CoV-2 entry and related mental disorders.

Despite the production of several SAR-CoV-2 vaccines, the SARS-CoV-2 virus will likely become endemic (Shaman and Galanti, 2020; Veldhoen and Simas, 2021). We, therefore, must study and develop an understanding of how infectious diseases like SARS-CoV-2 may contribute to long-term conditions such as mental health. The following review aims to highlight genes perturbed by a corona viral infection that are also implicated in mental disorders, emphasizing the effects of the SARS-CoV-2 virus. We begin by discussing host proteins vital to viral entry, a discussion of host proteins and factors that are affected downstream. Finally, we conclude by discussing how these host proteins relate to the etiology of mental health disorders (Figure 1).

## SARS-CoV-2 Structural Proteins and Their Roles in Viral Entry

SARS-CoV-2 exploits several proteins, including host proteases and host receptors, to gain entry to cells. The S protein, by which the virion enters host cells, must be cleaved by host proteases. Once the S protein has been primed, the protein can then bind host receptors, and the virion can then fuse with the host membrane. These host proteins vary in their spatial-temporal expression, but they each play a role in inflammatory responses, among other physiological effects. Understanding the functional functions of these entry proteins is crucial in understanding their role in the SARS-CoV-2 infection (Table 1).

**TABLE 1:** Host genes involved in SARS-CoV-2 infection and mental disorders.

Gene	Symptom/disorder	Summary	References
**Entry proteins**			
TMPRSS2	Depression	TMPRSS2 is implicated in depression associated with prostate cancer.	Rice et al., 2018; Wang et al., 2020
ADAM-10/17	Schizophrenia, depression, bipolar disorder, and conduct disorder	Increased levels of ADAM17 are associated with the diagnosis of schizophrenia in post-mortem brain tissue and CSF. A SNP located in ADAM10 was significantly associated with conduct disorder.	Jian et al., 2011; Qian et al., 2016; Hoseth et al., 2017
FURIN	Alzheimer’s disease, Schizophrenia	rs4702 was significantly associated with schizophrenia was detected both by GWAS and eQTL analyses. This SNP is also associated with reduced FURIN and BDNF expression.	Fromer et al., 2016; Hou et al., 2018
ACE2	Anxiety, depression, cognitive impairment	ACE2 is implicated in the dysregulation of the HPA axis following SARS-CoV-2 infection.	Steenblock et al., 2020
DPP4	PTSD, depression, other neuropsychiatric illnesses	NPY is a ligand for the DPP4 or CD26 receptor and has been a proposed biomarker for these illnesses.	Canneva et al., 2015; Gołyszny and Obuchowicz, 2019
XCR1	Traumatic brain injury	XCR1 expression increased significantly in the thalamus and hippocampus beginning 24 h post-injury	Ciechanowska et al., 2020
HMGB1	Schizophrenia	SCZ patients exhibited increased expression variability in HMGB1 and several other genes.	Huang et al., 2020
	Bipolar disorder	Serum levels of HMGB1 were significantly high in the bipolar patients compared to the controls.	Marie-Claire et al., 2019
Neuropilin	Major depressive disorder	Authors found increased NRP-1 expression in the post-mortem PFC samples from patients diagnosed with MDD than in controls.	Goswami et al., 2013
**Downstream of entry: inflammation**			
GSK-3β	Schizophrenia and bipolar disorder	Increased levels of GSK-3β were found in nasal biopsies of bipolar patients and the blood, serum, and CSF of patients with SCZ.	Narayan et al., 2014; Mohammadi et al., 2018
HLA	Schizophrenia	Several HLA genes, including HLA-A10, HLA-B, and HLA-DRB1, have been linked to SCZ	Carter, 2009
TLR (7/8)	Depression	Increased mRNA expression of TLR3 and TLR4 in the brains of depressed non-suicidal and suicidal subjects	Pandey et al., 2014
Interleukins	Autism Schizophrenia	IL-23 and IL-17 are implicated in immune dysregulation seen in patients with schizophrenia and experimental models of autism.	Debnath and Berk, 2017; Alves de Lima et al., 2020
CXCR6	Anxiety	Meningeal γδ T cells expressing CXCR6 were shown to influence anxiety in mice	Alves de Lima et al., 2020
CCR1	Bipolar and schizophrenia	Greater expression of CCR1 and 28 other genes were found in patients diagnosed with schizophrenia when compared to patients diagnosed with bipolar disorder	de Baumont et al., 2015
**Downstream of entry: cell survival**			
Histone complex H3.3	Depression	H3.3 was found to be elevated in the Nucleus Accumbens of depressed humans.	Lepack et al., 2016
SWI/SNF complex genes	Major depressive disorder and schizophrenia	The SWI/SN subunit, BRM (SMARCA2), has been associated with self-reported MDD and schizophrenia.	Amare et al., 2020; Wu et al., 2020a
ARID1A/B	Craniofacial abnormalities	Mutations in ARID1A are associated with craniofacial abnormalities, while mutations in ARID1B are associated with autism spectrum disorder and SCZ.	Son and Crabtree, 2014; Pagliaroli and Trizzino, 2021
BDNF	Schizophrenia	Decreased BDNF expression has been associated with schizophrenia.	Bar-Yosef et al., 2019; Suchanek-Raif et al., 2020
	Anxiety, Major Depressive Disorder	A common SNP of BDNF, rs62265, is a missense mutation that has been associated with anxiety, major depression and suicide, and neurodegenerative disease, as has dysregulation of mTOR signaling	Dincheva et al., 2016; Youssef et al., 2018
SLC6A20	Schizophrenia and schizoaffective disorder	Hyperprolinemia has been reported in conjunction with SCZ and schizoaffective disorder.	Jacquet et al., 2005; Clelland et al., 2011
**Downstream of entry: autophagy**			
FYCO1	Senescence	A significant decrease in FYCO1 expression was associated with senescence	Cheng et al., 2007
	Neurodegenerative disorders	FYCO1 is involved in the clearance of α-synuclein aggregates.	Saridaki et al., 2018
CTSB/L	Alzheimer’s disease Traumatic brain injury	Increased levels of cathepsin B in the cytosol, plasma, and CSF have been associated with cognitive dysfunction in Alzheimer’s and traumatic brain injury.	Hook et al., 2020
CALM/CaMKII	Schizophrenia	Calmodulin levels were reportedly altered in postmortem lysates taken from ACC, CC, and the temporal lobe in patients with schizophrenia.	Vidal-Domènech et al., 2020
**Downstream of entry: endocrine signaling**			
Estrogen receptor	Schizophrenia	Increased polymorphisms in ERα have been associated with SCZ. And circulating levels of estrogen have been associated with psychosis.	Min et al., 2012; Brzezinski-Sinai and Brzezinski, 2020
Androgen receptor	Depression Bipolar	Increased levels of AR expression were reported in patients with bipolar disorder.	
SHBG pathway	Depression	Positively and statistically significantly associated with depression risk (*p* = 0.003) in all women.	Colangelo et al., 2012
	Schizophrenia	In a study of schizophrenic male patients and a group of undiagnosed adults, both treated and untreated patients had lower serum levels of SHBG than undiagnosed controls.	Costa et al., 2006
**Mental disorders**			
TGF-beta	Schizophrenia and psychosis	TGF-Beta plays a role in the immune-inflammatory response and the compensatory immune-regulatory reflex system, which contribute to the etiology of schizophrenia.	Roomruangwong et al., 2020

### Host Proteases

The SARS-CoV-2 S protein must be primed by host proteases before it can bind to host receptors and infect cells. The host proteases that have been identified in helping aid in the binding of the S protein to host receptors include TMPRSS2, ADAM10/17, and Furin (Hussain et al., 2020). TMPRSS2 was found to increase viral entry into host cells significantly and is expressed in astrocytes and oligodendrocytes (Heurich et al., 2014; Dong et al., 2020). Previous work has demonstrated that camostat mesylate, a TMPRSS2 inhibitor, resulted in the blockage of SARS-CoV-2 into TMPRSS2^+^ cells (Hoffmann et al., 2020). ADAM-10/17 (A disintegrin and metalloprotease 10 and 17) are proteases that cleave the extracellular domain of ACE2. However, they are reportedly less efficient than TMPRSS2 (Heurich et al., 2014; Aljohmani and Yildiz, 2020). FURIN also aids SARS-CoV-2 entry. The FURIN protein is an endoprotease and is expressed in hippocampal and cortical neurons (Yang et al., 2018). FURIN cleaves proteins within a specific motif (R/K)-(2X) n-(R/K) and plays a role in priming the SARS-CoV at the S1/S2 site (Coutard et al., 2020). This cleavage allows the virus to shed the spike protein and enter the host cell. The use of the protease is thought to be a key component of the pathogenicity of many viruses, including SARS-CoV-2 infection (Coutard et al., 2020; Fitzgerald, 2020). Of the known pathogenic beta coronaviruses, only the SARS-CoV2, MERS-CoV, and HCoV-OC43 viruses possess the FURIN cleavage complex motif. This protease also plays a role in apoptosis, inflammation of the vasculature, and lipid metabolism (Liu et al., 2020).

### Essential Host Proteins That Interact With SARS-CoV-2

ACE2 is a part of the renin-angiotensin-aldosterone system (RAAS), and it is the principal host receptor used by SARS-CoV-2 (Motaghinejad and Gholami, 2020). The RAAS functions to maintain blood pressure by regulating fluid and electrolyte balance and vascular diameter (Wiese et al., 2020). The SARS-CoV-2 infection leads to the downregulation of ACE2, leading to what is referred to as Angiotensin II intoxication (Sfera et al., 2020; Wiese et al., 2020). ACE2 is expressed throughout the epithelia of the respiratory tract. However, the expression of ACE2 can be described as a gradient, where it is highest in the proximal nasal epithelia and attenuates as one proceeds to the epithelia of the lower respiratory tract (Hou et al., 2020). Within the central nervous system, the ACE2 receptor is expressed in both neurons and glial cells (Venkatakrishnan et al., 2020). It is also important to note that the expression of ACE2 and TMPRSS2 also increases with age, according to an investigation of temporal expression profiles in mice at ages 2 months and 2 years (Bilinska et al., 2020).

DPP4 is a ubiquitously expressed serine protease that plays a role in inflammation energy metabolism and has also been reported as a marker of senescence (Klemann et al., 2016; Kim et al., 2017; Shao et al., 2020; Rohmann et al., 2021). The DPP4 protein is widely expressed in many cell types throughout the CNS, including dopaminergic neurons, macrophages, and glia (Venkatakrishnan et al., 2020). Although it is primarily known as the host receptor utilized by the MERS-CoV virus, previous work has shown that SARS-CoV-2 may also use as a point of entry. A protein docking simulation and subsequent analysis of free energy binding found that SARS-CoV2 bound firmly to DPP4 (Li et al., 2020). It is worth noting that the RAAS system and the DPP4 receptor are dysregulated in diabetes, a risk factor in severe COVID illness (Valencia et al., 2020).

### Chemokine Receptors

Lymphopenia is one of the symptoms seen in patients with COVID-19. This observation has led to the notion that SARS-CoV-2 might also utilize other receptors, like XCR1, to facilitate T-Cell entry (Mobini et al., 2021). A structural study of binding affinity found that XCR1, in addition to several chemokine and immune receptors, had a higher binding affinity for the SARS-CoV-2 S protein than ACE2. XCR1 and other chemokine receptors are present in many types of immune cells. The XCR1 gene is upregulated in response to traumatic brain injury (Mobini et al., 2021). Several other chemokines, as well as their receptors, have been linked to prognostic outcomes in SARS-CoV and MERS-CoV infection (Khalil et al., 2021).

### Neuropilin

Neuropilin is a host receptor that concretizes the overlapping impacts of SARS-CoV-2 infection as it plays a role in the inflammatory response, angiogenesis, and nerve growth, as well as synaptogenesis (Cai and Reed, 1999; Mayi et al., 2021). Investigators who used x-ray crystallography were able to demonstrate that SARS-CoV-2 spike protein cleaved at the furin site was able to bind with neuropilin (NRP1) (Daly et al., 2020). Neuropilin is known to bind proteins cleaved by FURIN protease. In an investigation of host cell entry, the authors used HEK-293 T cells transfected with plasmids to permit the expression of ACE2 and NRP1 (Cantuti-Castelvetri et al., 2020). Furthermore, comparative analysis of postmortem olfactory epithelium from COVID-19 patients and uninfected controls showed that SARS-CoV-2 could infect NRP1 positive cells of the olfactory epithelium (Cantuti-Castelvetri et al., 2020). Although the levels of ACE2 in the cells of the olfactory epithelium were relatively low, the authors found that expression levels of high levels of NRP1 and oligodendrocyte transcription factor (OLIG2), a marker for neuronal progenitors of the olfactory tract, were higher by comparison (Cantuti-Castelvetri et al., 2020).

## Host Mechanisms Activated by SARS-CoV-2 Infection

Once SARS-CoV-2 begins to proliferate and spread, innate immunity is deployed as T lymphocytes, and dendritic cells are activated by pattern recognition receptors like toll-like receptors (TLRs) (Bai et al., 2021). However, this innate immunity is overcome by viral suppressors of RNAi (VSRs) (Bai et al., 2021). This leads to the release of inflammatory factors, which in severe cases may lead to a cytokine storm, resulting in tissue damage to organs such as the lungs and heart (Mortaz et al., 2020). These inflammatory factors and cytokines cause adaptive immune cell activation as CD4+ T-cells to act as antigen-presenting cells, and CD8+ T cells are deployed to kill infected cells (Mortaz et al., 2020). Viruses like SARS-CoV-2 have evolved methods of evading host immunity and usurping cellular machinery involved in cell survival, senescence, autophagy, mitophagy, etc., to enable their proliferation (Alcock and Masters, 2021). These mechanisms are further impacted by age as well as hormone signaling. In this section, we explore the effects of SARS-CoV-2 entry and genes involved in the downstream process (Table 1).

### Inflammation

The TLRs are molecular pattern recognition receptors that help to monitor the external cellular environment for pathogenic-associated molecular patterns (PAMPs) and damage-associated molecular patterns (DAMPS) (Lim and Staudt, 2013; Kumar, 2019; Liu et al., 2019a). The activation of TLRs following SARS-CoV-2 infection can incite a cytokine storm within the respiratory endothelia. However, it is also capable of activating glial cells of the CNS, releasing several inflammatory factors such as interleukin-1 (IL-1), IL-6, IL-12, C-X-C motif chemokine ligand 10 (CXCL10), C-C motif ligand 3 (CCL3), CCL5, CCL2, TNF-alpha, CXCR6, XCR1, and CCR1, causing chronic inflammation and brain damage (Bouças et al., 2020; Coperchini et al., 2020; Jakhmola et al., 2020; Wu et al., 2020b; Khanmohammadi and Rezaei, 2021). Several of these chemokines and inflammatory factors are expressed in astrocytes, glia, neurons, neural stem cells, and oligodendrocytes (Sowa and Tokarski, 2021). The cytokine storm, particularly the release of TNF-alpha, then leads to the suppression of B-cells and thus antibody production (Kumar et al., 2021). One host protein that is critically involved in the cytokine storm is GSK-3β.GSK-3β is a serine-threonine kinase involved in the inflammatory response to infectious disease and plays a role in the phosphorylation of the SARS-CoV-2 N-protein. Inhibition of GSK-3β by drugs such as lithium has been demonstrated to reduce viral replication and enhance immune response (Taylor et al., 2016; de Souza et al., 2020; Rana et al., 2021). Human Leukocyte Antigen (HLA) also plays a key role in genes regulating the immune response to pathogens through antigen presentation. However, the effect of HLA variants on susceptibility and resistance in coronavirus infection is less evident in the case of SAR-CoV-2 infection (Saulle et al., 2021). For example, the HLA-A*24:02 allele was reported to be both a contributing factor to susceptibility and resistance to SARS-CoV-2 infection in separate investigations (Saulle et al., 2021).

### Chromatin Remodeling

The pro-inflammatory High Mobility Group Box 1 (HMGB1) is a non-histone protein that also provides an entry point for SARS-CoV-2 (Andersson et al., 2020). HMGB1 is involved in organizing chromatin but acts as a damage signal when released by cells, such as neurons and glia, under conditions of stress or inflammation (Paudel et al., 2018). When necrotic cells release DAMP and PAMP molecules in the extracellular milieu, they can bind with HMGB1. These complexes of HMGB1 and DAMP and PAMP signals are then taken up by the cell through endocytosis and translocated to lysosomes. This activity leads to increased proinflammatory effects by breaking down the lysosomal membrane and releasing cytokines and other factors into the cytosol (Andersson et al., 2020). The extent to which chloroquine compounds may provide some benefit in COVID infections is that they might prevent the transfer of PAMPs and DAMPs containing SARS-CoV-2 RNA to the cytosol (Andersson et al., 2020).

Previous reports have demonstrated correlations between severe SARS-CoV-2 infection and cell cycle arrest in the S/G2 phase (Suryawanshi et al., 2021). For example, the C-terminus of the E-protein of the SARS-CoV and SARS-CoV-2 shares a very similar motif to the N-terminus of histone 3 (Gordon et al., 2020). Recently several proteins involved chromatin remodeling were identified in a genome-wide CRISPR screen in Vero-E6 cells infected with SARS-CoV-2, MERS-CoV, bat HKU5 expressing the SARS-CoV-1 S protein, and the vesicular stomatitis virus expressing the SARS-CoV-1 S protein. The authors found that AT-rich interactive domain-containing protein 1A (ARID1A) was a pro-viral gene in the case of infection by SARS-CoV-2 and MERS-CoV viruses (Wei et al., 2021). ARID1A/B is a component of the mammalian BRG1/BRM (BAF) complex, involved in chromatin remodeling and cell cycle arrest (Shigetomi et al., 2011; Pagliaroli and Trizzino, 2021). ARID1A is ubiquitously expressed in neural stem progenitor cells and throughout the brain (Liu et al., 2021). Another cellular component found to be perturbed by SARS-CoV-2 infection was the SWI/SNF (SWItch/Sucrose Non-Fermentable) complex, which is responsible for ATP-dependent chromatin remodeling. Interference with cell cycle progression allows the SARS-CoV-2 to hijack cellular machinery to increase viral replication (Kumar et al., 2021).

### Cell Survival

Bone-derived neurotrophic factor (BDNF) is a growth factor that plays a role in neurotransmission and neuroplasticity. It is expressed throughout the brain, including in astrocytes, Schwann cells, and neurons (Sakharnova and Vedunova, 2012). BDNF binds to tyrosine kinase B (Trk B), initiating a signal cascade that leads to the activation of the mechanistic target of rapamycin (mTOR), which promotes survival, growth, and differentiation of neurons (Bar-Yosef et al., 2019). SARS-CoV-2 has been demonstrated to enhance mTOR complex 1 (mTORC1) activity (Bar-Yosef et al., 2019). Calmodulin is not only an essential regulator of cellular activity, including apoptosis, neurotransmitter release, etc. (Yu et al., 2002; Ando et al., 2013; Schweitzer et al., 2021). Solute carrier family six-member 20 (SLC6A20) plays a role in the regulation of glycine as well as *N*-methyl-D-aspartate (NMDA) signaling (Bae et al., 2021).

### Senescence and Mitophagy

SARS-CoV-2, like many other viruses, is thought to induce senescence in host cells through the increased binding of Angiotensin II (ANGII) to the Angiotensin II Type 1 receptor. ANGII acts as a toxin with respect to the host’s cells’ mitochondria through activation of nicotinamide adenine dinucleotide phosphate (NADPH) oxidase and the creation of reactive oxygen species (ROS), H_2_O_s_ (Chang et al., 2020). This increase leads to the formation of hydroxyl radicals that cause DNA damage and the activation of poly ADP-ribose polymerases (PARPs), which are DNA damage sensors and deplete stores of NAD+ and exacerbate both the dysfunction of mitochondria. The depletion of NAD+ also results in the reduced mitophagy the increased formation of ROS, which in turn activates ADAM17 and inhibits nitric oxide (NO) synthesis (Dikalov and Nazarewicz, 2013; Chang et al., 2020; Sfera et al., 2020). ADAM17 is also a metalloprotease that has been reported to prime the SARS-CoV-2 spike protein (Heurich et al., 2014).

### Autophag*y*

Autophagy plays an essential role in the homeostatic balance between cell survival and cell death. Previous work has shown that coronaviruses MERS-CoV and SARS-CoV can prevent autophagosomes from binding to lysosomes (Randhawa et al., 2020). The SARS-CoV-2 infection has been shown to reduce zinc finger FYVE and coiled-coil domain-containing autophagy adaptor 1 (FYCO1) expression, which participates in autophagosome maturation through the Rab7 effector protein, a late endosomal GTPase (Cheng et al., 2007; Pankiv et al., 2010; Randhawa et al., 2020). FYCO1 is expressed in several different cell types within the cortex (Mestres et al., 2020). The cysteine proteases cathepsin B (CTSB) and cathepsin L (CTSL) have also been implicated, alongside TMPRSS2, in the activation of the S proteins of the SARS-CoV-1, SARS-Cov-2, and MERS-CoV coronaviruses. These proteases are found in endosomes/lysosomes and participate in autophagy and apoptosis (Pišlar et al., 2020). Cathepsins consists of serine, aspartic, and cysteine proteases and are ubiquitously expressed (Vidak et al., 2019). Although the cysteine cathepsins are primarily located within the lysosome, where the acidic environment maintains their stability, the excess secretion of cathepsins is associated with inflammatory responses and disease (Huang et al., 2006; Gomes et al., 2020; Pišlar et al., 2020). Previous research has demonstrated host cell entry of corona-pseudoviruses via CTSL dependent endocytosis, and cysteine protease inhibitors effectively blocked viral entry (Simmons et al., 2005, 2011; Zhou et al., 2011; Rabaan et al., 2017). Much like ACE2 and TMPRSS2, CTSB/L is enriched in the lungs (Darbani, 2020). However, the gene expression of the CTSB/L in the cortex and cerebellum was greater relative to the gene expression of ACE2 and TMPRSS2, which were nearly undetectable in the same tissue (Darbani, 2020).

### Endocrine Signaling

Testosterone levels have emerged as a risk factor for severe SARS-CoV-2 infection, and sex hormone signaling genes have been identified in previous investigations as potential targets in the treatment of SARS-CoV-2. Androgen receptors (ARs) are expressed through the CNS; however, the cortical expression of the AR is higher relative to other structures (Schumacher et al., 2021). The receptor influences the expression of ACE2 and TMPRSS2. Previous investigations of the effects of anti-androgenic drugs on the expression of genes related to the pathogenesis of SARS found that AR is a transcriptional regulator of ACE2, Furin, and TMPRSS2 (Samuel et al., 2020; Wambier et al., 2020). The TMPRSS2 gene is a target of the androgen receptor, which enhances transcription of TMPRSS2 (Clinckemalie et al., 2013; Samuel et al., 2020). It is, therefore, worth noting that hyperandrogenism in women has been associated with a greater risk of severe complications related to COVID-19 infection (Moradi et al., 2020). Previous investigations have demonstrated that the estrogen receptor (ER) is expressed by all neural cells and plays a role in resistance to infection and influences cytokine and macrophage activity (Seli and Arici, 2002; Villa et al., 2016). Interventions targeting estrogen and estradiol have been proposed as potential treatments for SARS-CoV-2 (Hussman, 2020). Sex-binding globulin (SHBG) is produced and secreted by the liver, and it binds sex hormones such as testosterone, and estrogen, thus regulating their levels in the bloodstream (Colangelo et al., 2012). An observational study of COVID-19 patients found lower SHBG levels in patients who died.

## Potential Mechanisms of SARS-CoV-2 Mediated Mental Disorders

Several of the host proteins genes that contribute to the pathobiology of SARS-CoV-2 infection, such as those involved in chromatin remodeling, are critical in the development of the central nervous system (Moffat et al., 2019; Torres-Berrío et al., 2019; Pagliaroli and Trizzino, 2021). Other host proteases and cellular receptors are involved in neurodevelopment, cellular proliferation, neurotransmitter release, sympathetic nervous system activation, neuroinflammation, etc. (Seidah, 2011). For example, factors involved in chromatin remodeling such as SWI/SNF and HMGB1 the SWI/SNF complex are important to embryonic and neurodevelopment. Dysfunction in genes associated with this complex are associated with neuropsychiatric disorders, neurodegenerative disorders, and intellectual disability (Marballi et al., 2014; Son and Crabtree, 2014; Vogel-Ciernia and Wood, 2014; Gozes, 2017; Paudel et al., 2018). Meanwhile, markers of neuroinflammation like XCR1 and CCXR1 are also implicated in stress, infection, and traumatic brain injury (Ciechanowska et al., 2020). These conditions lead to the presence of damage signals or antigens that can thereby be recognized by receptors such as toll-like receptors. The binding of these signal molecules then initiates signaling pathways, which lead to increased expression of inflammatory cytokines. This, in turn, leads to the activation of the hypothalamic-pituitary-adrenal (HPA) axis and sympathetic nervous system and the release of adrenaline, epinephrine, etc. (Canneva et al., 2015). The SARS-Cov-2 infection has also been known to trigger Guillain–Barre Syndrome, an autoimmune disorder characterized by demyelination of peripheral nerve axons (Toscano et al., 2020). Neuroinflammation and autoimmune disorders such as rheumatoid arthritis and celiac disease have been linked to mental health disorders such as BPD, SCZ, and psychosis (Eaton et al., 2010; Bergink et al., 2014; Dasdemir et al., 2016; Goldsmith et al., 2016; Hong et al., 2017; Milenkovic et al., 2019). A study of a large cohort of 3.57 million births linked to the Psychiatric Care Register in Denmark found that the relative risk for individuals diagnosed with an autoimmune disorder to be diagnosed with SCZ was 1.2 (Eaton et al., 2010). In this section, we examine the host factors that play critical roles in the etiology of mental disorders (Table 1).

### Anxiety, Depression, and Suicide

SARS-CoV-2 entry protein, ACE2, exert neuroinhibitory influence within brain regions such as the middle temporal gyrus and posterior cingulate cortex (Chen et al., 2020). Angiotensin (Ang) 1–7, a product of ACE2, decreases the synthesis and reuptake of noradrenaline and increases its uptake (Gironacci et al., 2014). ACE-2 and Mas protein regulate brain function and release neurotrophic factors, like BDNF (Steenblock et al., 2020). This factor has several critical roles, including the formation, development, and inhibition of degeneration of the neurons. It also plays a role in stabilizing mood and in cognitive function. Decreases in ACE-2 activity or expression have been known to disturb normal neurologic functions. This inhibition of ACE2 and subsequent decrease in BDNF leads to neurodegeneration and may cause mental disorders such as anxiety, depression, and cognitive impairment (Steenblock et al., 2020). It is important to note that the AR regulates the expression of ACE2 and TMPRSS2. Both AR and TMPRSS2 are implicated in prostate cancer, and some data suggests that there may be an association between prostate cancer and depression and anxiety (Newby et al., 2015; Rice et al., 2018; Wang et al., 2020).

Other host proteins such as neuropilin and DPP4 also relate to depressive symptoms. The expression of neuropilin in olfactory epithelia seems to be related to major mental disorders such as MDD. In one investigation, the authors found a significantly higher expression of NRP1 in post-mortem samples from the PFC of patients diagnosed with MDD when compared to controls (*p* < 0.001) (Goswami et al., 2013). Similarly, the expression of neuropeptide Y (NPY), a ligand for the DPP4 receptor, has been proposed as a biomarker for diagnosing PTSD, depression, and other neuropsychiatric illnesses (Canneva et al., 2015; Schmeltzer et al., 2016; Gołyszny and Obuchowicz, 2019). NPY has anxiolytic effects, and in an investigation, NPY immunoreactivity was significantly decreased in the cerebral spinal fluid (CSF) of unmedicated patients with persistent unipolar depression (Heilig et al., 2004). There is currently a clinical trial underway to investigate the value of Vildagliptin, an anti-diabetic drug, as adjunctive therapy to the SSRI, Escitalopram, and PDE3 inhibitor, Cilostazol, for the treatment of MDD (Clinical Trial ID: NCT04410341). It is also worth noting that cathepsins play a role in processing proneuropeptides like neuropeptide Y and have been found to be moderately associated with higher cognitive function following exercise training (Funkelstein et al., 2008, 2012; Moon et al., 2016).

Neuroinflammatory and immune responses are known to contribute to the development of mental disorders. One investigation of postmortem tissue taken from the dorsolateral prefrontal cortex (DLPFC) found increased mRNA expression of TLR3 and TLR4 and the increased presence of pro-inflammatory factors in the brains of depressed non-suicidal, and suicidal subjects (Pandey et al., 2014). Increased expression of TLR3 also results in reduced expression of disrupted in schizophrenia 1 (DISC1), which leads to aberrant neuronal morphology (Chen et al., 2017). In fact, previous research has shown that treatment with endotoxin to stimulate inflammatory cytokines or even treatment with inflammatory kinases themselves can lead to symptoms of depression in people who were previously undiagnosed (Bonaccorso et al., 2002; Anttila et al., 2018). Another investigation of the unfolded protein response in rats found increased expression of TLRs 2, 4, 7, and 9 as well as inflammatory cytokines within the hippocampus (Timberlake et al., 2019). In patients with hepatitis C, interferon-alpha (IFN-α) treatment can lead to clinical symptoms of depression, which can be alleviated by antidepressant therapy. This finding suggests that depression is caused by inflammation, and typical presentations of depression may have some similar underlying mechanisms.

Expression of inflammatory markers, such as chemokine CXCR6, by meningeal γδ T cells, has been shown to influence anxiety in mice (Alves de Lima et al., 2020). Mice deficient in CXCR6 have been demonstrated to have fewer γδ T cells than controls. The γδ T cells, in turn, release IL-17, a gene implicated in autism spectrum disorder (ASD) and SCZ (Debnath and Berk, 2017; Alves de Lima et al., 2020). γδ T cell-deficient mice demonstrated reduced anxiety behavior in the open field test. The authors showed that these cells could control anxiety behavior through IL-17 signaling (Alves de Lima et al., 2020).

Sex hormones and neuroimmune responses play converging roles in the etiology of mental disorders (Kokkosis and Tsirka, 2020). Lower testosterone is a predictor of depression symptoms in men, while higher levers of free testosterone in serum have been linked to manic episodes in men (Ozcan and Banoglu, 2003; Sankar and Hampson, 2012). Anti-androgenic therapies have been considered as a potential treatment for vulnerable populations (Bravaccini et al., 2021). A greater risk of depressive symptoms was positively associated with SHBG in a study of depressive disorders in post-menopausal women. SHBG was positively and statistically associated with depression risk (*p* = 0.003) in all women (Colangelo et al., 2012). In a study of schizophrenic male patients and a group of undiagnosed adults, the authors found that both treated and untreated patients had lower serum levels of SHBG (33.3 and 26.6 nmol/L) than undiagnosed controls (48.4 nmol/L, *p* < 0.05) (Costa et al., 2006).

### Bipolar Disorder, Schizophrenia, and Psychosis

Several infections, such as cytomegalovirus, herpes simplex virus, and parasitic infection by *T. gondii*, have been noted for interacting with the HLA system and for their association with affective disorders like BPD and SCZ (Parks et al., 2018). SCZ has been linked to several polymorphisms in the major histocompatibility complex (MHC) or the HLA system through several GWAS (Parks et al., 2018). HLA genes are expressed in astrocytes and microglia within the brain, although primarily in microglia (Tian et al., 2012). Previous investigations have identified HLA-B*4601, HLA-B*0703, HLA-B*4601, HLA-C*0801, and HLA-DRB1*1202 as alleles associated with severe illness following SARS-CoV-2 infection (Lin et al., 2003; Ng et al., 2004; Morsy and Morsy, 2021). The HLA-DRB*0301 and HLA-Cw*1502 alleles were associated with a reduced frequency of severe infection. These clusters, namely HLA-B, HLA-DRB1, HLA-C, HLA-DRA, HLA-DQA, HLA-DQB, HLA-DPB, have been associated with mental health disorders, i.e., SCZ, BPD, and PTSD (Carter, 2009). A gene-wide association study of 13,4982 cases and 663 controls found significant associations between the Notch 4 intronic variant rs3131296 and HLA alleles: HLA-DRB1*0301 and HLA-B*0801 (*R*^2^ = 0.86 and 0.81, respectively) (Stefansson et al., 2009). In a separate investigation of molecular pathways underlying SCZ and BPD, the authors found that patients demonstrate more variation in the HLA-C and HLA-DRA genes than would be expected by chance (Marco et al., 2015). It is also worth noting that PTSD has also been found to be associated with HLA alleles (HLA-B*5801, HLA-C*0701, HLA-DQA1*0101, HLA-DQB1*0501, and HLA-DPB1*1701) in a case-control study of 403 diagnosed patients with 369 individuals who had been exposed to trauma (Katrinli et al., 2019).

Differential levels of cytokines and cytokine receptors have been found between the first episode, acute relapse of psychosis, and post-treatment patients diagnosed with SCZ (Capuzzi et al., 2017). A previous meta-analysis of first-episode psychosis, acute relapse, and post-treatment SCZ patients compared effect sizes of blood levels of inflammatory markers (cytokines, cytokine receptors, and antagonists) (Miller et al., 2011). Significant differences between the effect sizes of several inflammatory markers were found between post-treatment patients diagnosed with SCZ and first-episode psychosis and acutely relapsed patients (Miller et al., 2011). These cytokines and receptors include including IL-6, IL-12, TNF-α, IL-1β, IL-8, transforming growth factor-β (TGF-β), IL-1RA, IFN-γ, sIL-2R, and IL-10 (Miller et al., 2011). It is worth noting that levels of IL-6 and TNF-α were significantly correlated with survival in SARS-CoV-2 in a previous investigation (Del Valle et al., 2020). Increased levels of circulating IL-1β, IL-12, IL-6, CXCL10, CCL2 have been reported in severe cases of SARS-CoV-2 infection (Coperchini et al., 2020). TGF-beta plays a role in regulating immune response and plays a role in the development of mental disorders like SCZ and symptoms such as psychosis (Sanjabi et al., 2017; Roomruangwong et al., 2020). Increased expression of chemokines such as CCR1 has been shown in postmortem brain tissue in patients diagnosed with SCZ compared to patients diagnosed with BPD (de Baumont et al., 2015).

Metalloproteases ADAM10/17 have been implicated in neurodegenerative disorders. They play a role in the proteolysis of the amyloid precursor protein (APP) and several other proteins (Vincent and Govitrapong, 2011; Qian et al., 2016). Similarly, another protein affected by SARS-CoV-2 infection, FYCO1, has been linked to neurodegenerative disorders, neuropsychiatric disorders, and senescence in age-accelerated mice (Cheng et al., 2007; Saridaki et al., 2018). However, ADAM10/17 have also been linked to SCZ, depression, BPD, and conduct disorder, a condition that has been found to be comorbid with mood disorders (Jian et al., 2011; Marballi et al., 2012; Qian et al., 2016; Hoseth et al., 2017; Yuan et al., 2017; Pantazopoulos et al., 2020). A family-based association study found 20 variants associated significantly associated with conduct disorder; among these single nucleotide polymorphisms (SNPs), rs383902 was located within ADAM10 (*p* = 0.00036) (Jian et al., 2011). In one investigation of postmortem brain tissue from BA9, using ANCOVA analysis, investigators found a significant difference in ADAM17 expression between the control and bipolar groups and levels observed in the schizophrenic group (*p* < 0.007). The authors also found a significant negative correlation between levels of neuregulin-1 (NRG-1) and ADAM17 in Broca’s area 9 samples taken from the post-mortem tissues of patients diagnosed with SCZ (Marballi et al., 2012). Similarly, Hoseth et al. (2017) found greater mRNA expression of ADAM17 in the plasma of SCZ patients vs. that seen in controls (Hoseth et al., 2017). ADAM10/17 influences glutamatergic signaling, which is also impacted by the SLC6A20 transporter protein. In a GWAS of NMDA receptors and the detection of their coagonists in cerebrospinal fluid, the authors found that a missense variant in SLC6S20 as associated with increased L-proline levels in CSF, thus demonstrating that SLC6A20 plays a role in the trafficking of proline to the CSF (Luykx et al., 2015). Hyperprolinemia has been previously reported in conjunction with SCZ and schizoaffective disorder (Jacquet et al., 2005; Clelland et al., 2011).

Neurotransmission may also be related to the expression of SARS-CoV-2 entry protease FURIN. FURIN was found among several genes linked to comorbidity SCZ and cardiometabolic illness, which gives insight into the etiology of these conditions (Liu et al., 2020). Several studies underscore the importance of furin in the CNS, as it has been linked to Alzheimer’s disease (AD) and SCZ (Scamuffa et al., 2006; Schrode et al., 2019; Yang et al., 2020). In a GWAS of 49 ancestry matched non-overlapping case-controls and 1,235 parent affected offspring trios, the authors found 108 loci that were significantly associated with SCZ (Schizophrenia Working Group of the Psychiatric Genomics Consortium, 2014). Of those, Fromer et al. (2016) found nineteen of the SCZ risk loci were enriched for eQTLs. However, only eight involved a single gene; among them was the gene encoding furin protease. The authors found that furin expression was downregulated by the risk variant rs4702 (GG to AA allelic conversion), a 3′ UTR variant, which was both the most significant SNP detected by GWAS and eQTL analyses (Fromer et al., 2016). The rs4702 SNP results in the alteration in the binding site for miR-338-3p. miR-338-3p is an mRNA that is expressed predominantly in mature neurons within the dentate gyrus (Howe et al., 2017). The authors noted that cells in which miR-338-3p was effectively knocked down showed aberrations in the number of primary dendrites as well as the angles of their extension from the soma (Howe et al., 2017). Interestingly enough, the rs4702 variant, which is associated with SCZ, may be protective against SARS-CoV-2 infection, as cells expressing rs4702 had reduced levels of vRNA relative to cells expressing the normative allele (AA) (Dobrindt et al., 2021). In a separate investigation, the rs4702 specific reduction in the expression of FURIN and BDNF was “mediated” by miR-338-3p (Hou et al., 2018). BDNF is a member of the BDNF-mTORC1, which helps to regulate synaptic plasticity, glutamatergic signaling, monoaminergic signaling, and autophagy. The SARS-CoV-2 infection has been demonstrated to increase the activity of mTORC in Vero kidney epithelial cells 24 h post-infection (Mullen et al., 2021). A common SNP of BDNF, rs62265, is a missense mutation that has been associated with anxiety, major depression, suicide, and neurodegenerative disease, as has dysregulation of mTOR signaling (Dincheva et al., 2016; Youssef et al., 2018; Bar-Yosef et al., 2019). Additionally, decreased BDNF expression has also been associated with SCZ (Suchanek-Raif et al., 2020). Epigenetic regulation of BDNF has also been demonstrated to play a role in mental illness, as methylation of genes associated with SCZ, like BDNF, has been linked to psychosis (Gavin et al., 2010).

Increased levels of GSK-3β in blood, serum, and CSF have been associated with SCZ (Mohammadi et al., 2018). The GSK-3β inhibitor lithium, which is utilized as a treatment for psychiatric disorders such as SCZ and BPD, has been shown to inhibit infection by several viruses, including coronaviruses (Murru et al., 2020). One investigation examined gene expression in 12 BPD patients and ten controls following two laser microdissections of the olfactory epithelia: one pretreatment with lithium in the second after 6 weeks of daily lithium treatment. The BPD patients demonstrated greater levels of GSK-3β than controls (Narayan et al., 2014). Lithium has been shown to inhibit GSK-3β, and similar to those studies the authors found that GSK-3β was reduced in the second microdissection samples taken from BPD patients following 6 weeks of daily lithium treatment (Harrison et al., 2007; Narayan et al., 2014; Zhao et al., 2017).

Genes responsible for chromatin remodeling are implicated in SCZ as well. The SWI/SNF complex protein ARID1A is typically associated with craniofacial abnormalities. However, mutations in the ARID1B gene have been associated with intellectual disability, ASD, and SCZ as well (Son and Crabtree, 2014; Pagliaroli and Trizzino, 2021). SWI/SNF-related matrix-associated actin-dependent regulator of chromatin, family a, member 2 gene (SMARCA2), encoding the SWI/SNF subunit, Brahma (BRM), has been associated with self-reported MDD and SCZ (Amare et al., 2020; Wu et al., 2020a). In an investigation of bivariate analyses of genome-wide association study results relating to depression combined with MDD, BPD, and SCZ, the authors found that the SMARCA2 gene and the SWI/SNF gene set were enriched. This indicated the role of epigenetic mechanisms in the etiology of complex mental health disorders (Amare et al., 2020). In a separate investigation, drugs capable of inducing psychosis were found to reduce BRM expression, while anti-psychotics led to increased expression of BRM (Koga et al., 2009).

Further demonstrating the importance of chromatin remodeling proteins in the etiology underlying SCZ is the HMGB1 protein. An investigation of gene expression in post-mortem tissue from 212 patients with SCZ and 214 undiagnosed controls found 87 genes demonstrated expression variability, including HMGB1 (Huang et al., 2020). In a separate study, serum levels of HMGB1 were significantly higher in bipolar patients than in undiagnosed controls. The authors found that serum levels of HMGB1 were significantly higher in the bipolar patients compared to the controls137. A systematic review of the literature also found increased levels of HMGB1 in conjunction with several studies of mouse models of depression (Zhang et al., 2019). One drug, minocycline, was found to reduce depressive-like symptoms in a mouse model of depression. This reduction was associated with a significant decrease in the translocation of HGMB1 from neurons and microglia (Wang et al., 2020).

Sex hormones have also been shown to play a role in the risk of developing SCZ (Kokkosis and Tsirka, 2020). Women with polycystic ovarian syndrome (PCOS) have also been demonstrated to be at greater risk of developing psychiatric disorders such as bipolar disorder and SCZ (Owens et al., 2019). An investigation of androgen receptor expression among individuals diagnosed with SCZ, BPD, and undiagnosed controls (*n* = 35, 31, and 34, respectively) found increased expression of AR among individuals diagnosed with bipolar disorder relative to individuals diagnosed with SCZ and control volunteers. No significant differences were observed in 5α-reductase between the experimental groups. However, a small but significant correlation was found between bipolar disorder and 5α-reductase expression (*r* = 0.422, *p* < 0.01) (Owens et al., 2019). Hormones have been demonstrated to affect neuropeptides involved in stress and anxiety, like oxytocin and corticotropin-releasing hormone (CRH) (Wang and Wang, 2021). Previous research has linked decreased oxytocin and oxytocin receptor levels to first-episode SCZ and bipolar II disorder (Liu et al., 2019b; Wei et al., 2020). The therapeutic use of OXT has been proposed as a treatment to protect against cardiovascular damage caused by SARS-CoV-2 infection (Wang and Wang, 2021).

## Discussion

Several host genes affected by SARS-CoV-2 infection are implicated in mental disorders and neuropsychiatric symptoms. Of the host genes perturbed by the coronavirus spike protein, many are involved in innate and adaptive immunity, stress response, cell cycle regulation, and other biological functions. These genes have also been implicated in mental disorders such as depression, SCZ, and bipolar disorder. Other components of the SARS-CoV-2 virion, such as E and N proteins on host proteins PSD95 and RIG-1, also relate to neuropsychiatric symptoms (Javorsky et al., 2021; Oh and Shin, 2021).

Several other genes that are dysregulated in mental disorders, such as DISC1, phosphodiesterase 4B (PDE4B), and neurexin-1 (NRXN1), could also be impacted by SARS-CoV-2 infection and contribute to neurotropism and inflammation in the CNS. We previously noted that increased TLR3 signaling leads to reduced DISC1 expression and aberrant neurogenesis. A recent transcriptomics study of publicly available datasets demonstrated that DISC1 is downregulated by COVID-19 (Alqutami et al., 2021). Though the exact role that DISC1 plays in complex mental disorders is unclear, DISC1 is an important component in the formation of the immune synapse. DISC1 forms a complex with Girdin and dynein that allows for the translocation of the microtubule-organizing center to the synapse; however, in DISC1 knock-out cell lines, the MTOC fails to translocate to the immune synapse (Maskalenko et al., 2020). The DISC1 pathway is a massive multi-step pathway of 203 genes that can be subdivided in the interactome and regulome (Teng et al., 2017). DISC1 and the DISC1 pathway genes like PDE4B and NRXN1 are implicated in several mental health disorders (Millar et al., 2007; Korth, 2009; Hu et al., 2019). PDE4B is found in the DISC1 interactome, and differential expression of PDE4B has also been noted in relation to COVID-19 infection (Alqutami et al., 2021). PDE4B has been shown to regulate cytokine signaling pathways (Lugnier et al., 2021; Moolamalla et al., 2021). Several adjunct therapies for the treatment of SARS-CoV-2 symptoms have been identified that target PDE4B (Lugnier et al., 2021; Moolamalla et al., 2021). Studies of microRNAs as potential targets of treatments for viral infection have shown that miR-1290 is upregulated in SARS-CoV-2 infection, and this is predicted to result in downregulation of NRXN1 expression (Chen and Wang, 2020; Guterres et al., 2020).

Long-lasting pulmonary symptoms, pain, fatigue, and other symptoms stemming from coronavirus infection have been documented throughout the literature. However, currently, no studies have investigated the mechanisms concerning the long-lasting mental health symptoms or disorders that might result from COVID-19 infection. However, several publications have enumerated observations of long-COVID neuropsychiatric symptoms and life stressors that affect mental health (Crook et al., 2021). One published review listed several probable risk factors related to PTSD and psychological dysfunction, including isolation, loss of a loved one, disability, and occupation (Boyraz, 2020). An investigation of1,427 United States adults reported the percentage of respondents reporting depressive symptoms increased from 27.8% in early 2020 to 32.8% just 1 year later (Ettman et al., 2021).

It is unclear what the precise causes of long COVID or neuropsychiatric symptoms could be the result of neuroinvasion by coronavirus in the brain and CNS or could result from systemic inflammation or a combination of both. There are conflicting studies regarding the specific ability of coronaviruses to cross the blood-brain barrier and infect the CNS or to be transmitted from neuron to neuron via the olfactory bulb (Thye et al., 2022). However, clinical observations of anosmia and encephalitis would suggest that SARS-CoV-2 and other coronaviruses are capable of both (Mondelli and Pariante, 2021). The precise mechanisms leading to long-term psychological sequelae are yet elusive. Some investigators have concluded that there may be myriad factors contributing to long COVID cases, including prolonged inflammation, ischemia, neuroinvasion, prolonged sedation, etc. (Alonso-Lana et al., 2020; Song et al., 2020). Given the comorbidity between autoimmune disorders and mental disorders and observations of increased levels of pro-inflammatory factors in the absence of encephalitis, it seems that inflammation is likely the underlying cause (Soria et al., 2018; Alonso-Lana et al., 2020; Proal and VanElzakker, 2021).

Several animal models suitable to the study of COVID-19 are currently available. Among these models are rhesus macaques, ferrets, mice expressing the human ACE2 receptor, and Golden Syrian hamsters (Jia et al., 2021). All these animal models feature pathological symptoms related to human pathological symptoms encountered with COVID-19. These symptoms include mild to moderate pneumonia, increased inflammatory markers, and weight loss. However, only two of these models are commonly utilized to investigate behavioral phenotypes: rhesus macaques and mice. Rhesus macaques are animal models that are used to investigate mental disorders such as anxiety. Many studies utilize transgenic mice to investigate obsessive-compulsive disorder (OCD), depression, SCZ, and ASD.

In this review, we summarized several host factors and pathways that are involved in coronavirus infection and are also implicated in neuropsychiatric symptoms. Though several of these host factors are expressed in the CNS, we have also provided evidence that their influence on widespread systemic inflammation may play a significant role in the development of long-term psychological symptoms stemming from COVID-19 infection. We’ve highlighted several cellular mechanisms that are impacted by SARS-CoV-2 infection and connected them to complex mental disorders such as MDD, SCZ, and BPD. We have elucidated the connection between DISC1 and DISC1 pathway proteins such as NRXN1 and PDE4B to viral infection as well as to mental disorders.

Future work should focus on the mechanisms by which infectious diseases like COVID-19 may impact mental illnesses of neuropsychiatric symptoms. This knowledge could contribute to interventions to lessen the effects of infection on the central nervous system or inform the development of treatments for existing mental disorders. Some of the host factors described here are already being investigated for their potential use as therapies or co-therapies for mental illness symptoms. However, further investigation is necessary to determine what impact coronavirus and other flu-like infections may have on mental symptoms and disorders. These investigations could elucidate the biological changes underlying the etiology of complex mental illnesses like SCZ, BPD, and depression.

## Author Contributions

RR and ST: conceptualization and writing—review and editing. RR, SS, CJ, and ST: writing—original draft preparation. ST: supervision and funding acquisition. All authors contributed to the article and approved the submitted version.

## Conflict of Interest

The authors declare that the research was conducted in the absence of any commercial or financial relationships that could be construed as a potential conflict of interest.

## Publisher’s Note

All claims expressed in this article are solely those of the authors and do not necessarily represent those of their affiliated organizations, or those of the publisher, the editors and the reviewers. Any product that may be evaluated in this article, or claim that may be made by its manufacturer, is not guaranteed or endorsed by the publisher.
